# Factors affecting length of stay in Children Hospital in Southern Iran

**DOI:** 10.1186/s12913-019-4799-1

**Published:** 2019-12-10

**Authors:** Tayebeh Baniasadi, Kobra Kahnouji, Nasrin Davaridolatabadi, Saeed Hosseini Teshnizi

**Affiliations:** 1 Student in Medical Informatics, Health Information Management Department, the school of Allied Medical Sciences, Tehran University of Medical Sciences, Tehran, Iran; 2 Social Determinants in Health Promotion Research Center, Hormozgan Health Institute, Hormozgan University of Medical Sciences, Bandar Abbas, Iran; 3 Associate Professor of Health Information Management, Fertility and Infertility Research Center, Hormozgan University of Medical Sciences, Bandar Abbas, Iran; 4 Assistant Professor of Biostatistics, Cardiovascular Research Center, Hormozgan University of Medical Sciences, Bandar Abbas, Iran

**Keywords:** Length of stay, Hospital, Patient

## Abstract

**Background:**

One of the effective indicators for determining the efficiency and optimal use of hospital resources is the length of stay (LOS). This study aimed to determine the patients’ length of stay and the factors affecting the LOS in the Children’s hospital.

**Method:**

A cross-sectional study was performed on Children Hospital medical record database including 350 records (April 2015 to Dec 2015). Records were selected by stratified random sampling with proportional allocation. Then the predetermined demographic and hospital variables were extracted through the study of patients’ medical records. All statistical analysis were performed using SPSS software.

**Results:**

The overall median of the LOS in the studied hospital was 3 days (IQR =3). The results showed that in this hospital the LOS has a significant relationship with the variables of time of admission, the place of residence, type of admission, and the degree of attending physician. Also, with the increasing number of visits, ultrasonography, counseling and laboratory test, LOS was increased.

**Conclusion:**

Improving processes related to diagnostic procedures, providing adequate staffing for specialized services in all hours of the day, preventing unnecessary and non-emergency admissions in the evening and night, will be effective in optimizing patient LOS.

## Background

Healthcare is one of the basic needs of every community. Since paying attention to health care and investment in this field increases labor productivity and increases production, it is important that in this part, sufficient resources be allocated and used optimally [1].

With the rapid increase in health care costs, the government and other healthcare providers have sought a solution to control costs and evaluate the performance of healthcare providing system. Therefore, the optimum use of health services has become an important goal [2].

One of the helpful indicators that can be used to manage hospital care, quality control, availability of hospital services, hospital policy and planning, as well as determine the efficiency and use of hospital resources, is the patients’ length of stay in the hospital [1].

Efficiency and productivity are essential for the economies of the countries and the survival of the organizations and institutions. Hospitals are forced to adopt strategies for the efficient use of resources to overcome the challenge of increasing costs [3]. Considering the important role of the hospital in the healthcare system and the fact that 40% of the health budget in developed countries and 80% in developing countries is allocated to the hospital, so more attention should be placed on regular evaluating and monitoring of hospitals performance indicators. Determining factors affecting each of these indicators can also play an important role in management decisions [4].

LOS as a key performance indicator is defined interval between admission and discharge of a hospitalized patient [3].

Studies have been done in the past, each of which describes a group of variables as influential factors on the patients’ length of stay in the hospitals [5].

There is no comprehensive agreement on the factors affecting the length of stay of the patient. Nevertheless, factors can divide into the following categories.

- Patient’s demographic variables such as age, sex, occupation, place of residence, and marital status
- Clinical variables such as the number of previous admissions, the cause of referral, and the cause of admission
- Management variables include admission on different days of the week, type of insurance, type of admission, and type of payment
- Hospital variables including hospitalization ward, the specialty of the physician, and academic degree of the physician [6].

Investigating and analyzing the length of hospital stay will be helpful for hospital management, especially in determining priorities, improving services and allocating resources appropriately according to the differences in the length of stay of different patients and demographic factors [1]. Therefore, the present study was conducted to determine the length of stay and the factors affecting on the LOS in the Children Hospital of Hormozgan University of Medical Sciences.

## Methods

This descriptive-analytic study was conducted in a cross-sectional design. We assessed the inpatients’ medical records admitted to Children’s hospital between” April 2015 to December 2015. This hospital is a specialty teaching hospital in Bandar Abbas, capital of Hormozgan province, south of Iran where patients from all over the province are admitted. Hospital wards include neonates, emergency, surgery, oncology, NICU, ICU, pediatric I, pediatric II, and pediatric III. Nephrology, Neurology and gastroenterology patients older than 28 days are admitted to Pediatric I ward. Asthma and allergy, endocrinology and cardiology patients are admitted in Pediatric II ward. General pediatric patients are admitted to Pediatric III ward and the attending physicians are board-certified pediatricians. In this study, the NICU, ICU, and the oncology ward admissions are not included. The number of inpatients admitted to other wards from April 2015 to December 2015 was 6265 patients. Calculated sample size using Cochrane formula and considering α = 0.05, the standard deviation of 4.34 for the mean of LOS and d = 0.5 [1], was 276 records.

To achieve greater accuracy, 350 inpatients medical records were selected by stratified random sampling with proportional allocation. Of which 84 records from the neonatal ward, 120 from pediatric I, 116 from pediatric II, 9 records from pediatric III, and 20 records from surgery ward have been selected.

The required data was extracted from patients’ records using a data collection sheet. Based on the literature review and key factors from similar studies, the checklist was prepared. Finally, the content validity of checklist was confirmed by experts of health information management and hospital management team. Data collection sheet included variables such gender, age, and patient’s residence as demographic characteristics, in addition hospital variables such as day, time, and type of admission, attending physician, duration of hospital stay, etc. Also, some of extracted quantitative variables included number of surgeries, diagnostic services, and clinical counseling. Data were collected through study of patient’ paper records and in some cases, using the Hospital Information System (HIS), via the presence of the researcher in the medical records unit of the hospital.

In this study, admission time from 7 am to 2 pm was categorized as morning admission, from 2 pm to 7 pm as evening admission and 7 pm to 7 am as night admission (same as staff work shifts) which differ in presence of pediatrician or subspecialist physician.

The qualitative variables were described with the number (n), percentage (%) and also quantitative variables were described using median and inter quartile range (IQR). The relationships between the LOS and nineteen factors were studied using nonparametric tests such as Mann-Whitney, Kruskal-Wallis, and Spearman correlation. For all analysis *p* < 0.05 was considered to be statistically significant. All statistical analysis was performed using SPSS software (IBM SPSS Statistics for Windows, Version 23.0. Armonk, NY: IBM Corp; 2015).

## Results

The median length of stay in the children’s hospital was 3 days (IQR =3). Minimum and maximum of LOS were one and 27 days respectively. 25% of patients stayed less than 2 days and 25% of them stayed in the hospital for more than 5 days. The median for age was 270 days (IQR = 700), of which the least was 1 day and the highest was 4747 days.

The results showed that between the length of stay and the place of residence and home town variables was a significant difference (*P* < 0.05) (Table 1). According to Table 2, there was also a significant relationship between the LOS and the time of admission, type of admission, and attending physician’s degree (*P* < 0.05). So resident outside Bandar Abbas, admission in the evening and night, rural residents, referred from other hospitals, and patients of a sub-specialist attending physician were associated with increased length of stay.

**Table 1:** Comparison of the mean LOS based on demographic characteristics

Characteristics		n (%)	Median (IQR)	*P*-value
Gender	Male	203 (58)	3 (4–2)	0.337
	Female	147 (42)	3 (5–2)	
Place of residence	Urban	240 (68.6)	3 (4–2)	0.002
	Village	110 (31.4)	3 (6–2)	
Home town	Bandar Abbas	208 (59.4)	3 (4–2)	0.003
	Outside Bandar Abbas	141 (40.3)	3 (5–2)	

*IQR* Inter Quartile Range

Missing data not entered in the table. So different numbers in the table are related to missing data

**Table 2:** Comparison of the mean LOS based on hospital variables

Characteristics		n(%)	Median (IQR)	*P*-value
medical insurance coverage type	Uninsured Patients	37 (11.2)	4 (6–2)	0.148^b^
	Social Security Insurance	138 (41.9)	3 (4–2)	
	Health Services Insurance	114 (34.6)	3 (5–2)	
	Forces Insurance	22 (6.7)	3 (5–2)	
	Iranian Health Insurance	18 (5.5)	2 (3–2)	
Day of admission	Holiday	41 (11.7)	3 (4–2)	0.703^a^
	Non-holiday	308 (88.3)	3 (5–2)	
Time of admission	morning (07:00 to 14: 00)	97 (27.7)	3 (4–2)	0.043^b^
	evening (14: 00 to 19:00)	95 (27.1)	3 (5–2)	
	night (19: 00 to 07: 00)	158 (45.1)	3 (5–2)	
Type of admission	Elective	63 (19.3)	2 (4–2)	0.038^b^
	Emergency and other wards	260 (79.5)	3 (5–3)	
	From other hospitals	4 (1.2)	6 (3–0)	
Inpatients wards	Neonatal	84 (24.1)	2 (6–2)	0.856^b^
	Pediatrics 2	116 (33.2)	3 (4–2)	
	Pediatrics 1	120 (34.4)	3 (4–2)	
	Pediatrics 3	9 (2.6)	2 (3–2)	
	Surgery	20 (5.7)	3 (5–1)	
Admitting Physician’s degree	GP	3 (0.9)	3 (3–3)	0.836^b^
	Resident	80 (22.9)	3 (5–2)	
	Specialist	187 (53.6)	3 (4–2)	
	Sub-Specialist	79 (22.6)	3 (4–2)	
Attending Physician’s degree	Specialist	45 (12.9)	2 (2–1)	0.010a
	Sub-Specialist	304 (87.1)	3 (5–2)	
Physician’s specialty	Neonatal	74 (21.2)	2.5 (6–2)	0.152^b^
	Neonatal cardiologist	36 (10.3)	3 (4–2)	
	Neonatal gastroenterologist	24 (6.9)	2.5 (4–2)	
	Pediatrician	48 (13.8)	2.5 (3–2)	
	Allergist	43 (12.3)	3 (5–2)	
	Endocrinologist	12 (3.4)	4 (4.75–2)	
	Nephrologist	51 (14.6)	3 (4–2)	
	Neurologist	24 (6.9)	2 (1–1)	
	hematologist	3 (0.9)	3 (2–2)	
	Pediatric Infectious Disease Specialist	26 (7.4)	2.5 (8–1)	
	General Surgeon	8 (2.3)	1 (1–1)	
Patients’ status at discharge	Recovery	7 (2)	2 (3–2)	0.479^b^
	Relative recovery	258 (75.4)	3 (4–2)	
	Discharge Without Physician’s Order	65 (19)	2 (5–2)	
	Need to Follow up after Discharge	11 (3.1)	2 (3–2)	

*IQR* Inter Quartile Range, ^a^Mann-Whitney test, ^b^ Kruskal-Wallis test

Missing data not entered in the table. So different numbers in the table are related to missing data

The LOS between medical insurance coverage types, inpatient wards, and patient status at discharge was not significantly different (*P* > 0.05).

According to the result of Spearman correlation test with the increasing number of laboratory test, counseling, ultrasonography, and visit the length of stay in the hospital was increased significantly (*p* < 0.001) (Table 3).

**Table 3:** Relationship between the quantitative variables and LOS

Characteristics	Mean ± SD	Spearman correlation coefficient	*P*-value
Age	592.22 ± 873.25	−0.082	0.140
Number of previous hospitalizations	0.34 ± 0.86	0.108	0.047
Number of laboratory tests	6.70 ± 6.65	0.434	< 0.001
Number of ultrasonography	0.22 ± 0.51	0.183	0.001
Number of medical counseling	0.51 ± 1.31	0.300	< 0.001
Number of visits	12.72 ± 11.19	0.655	< 0.001
Number of surgeries	0.05 ± 0.25	−0.023	0.669

## Discussion

According to the results of this study, patients who had been admitted in the evening (14,00 to 19,00) and night (19,00 to 07,00) had a longer LOS. It seems that the absence of specialized doctors and the failure to provide some diagnostic and therapeutic services at these hours were the reason for the increased length of stay. Bekmezian et al. found that children who were admitted at the emergency department between midnight and 8 am, had a longer LOS [7].

Perhaps ensuring that patient care and modifying care processes especially the presence of specialist doctors, can be considered as a means of preventing an increase in the average length of stay. Also, using capabilities of information and communications technology (ICT) such as telemedicine and tele-counseling with a well-trained and qualified medical team will be of great help. In this regard, optimal use of available and low-cost devices such as smartphones or web-based applications can be a solution to these problems by providing tele-counseling and remote visit without the presence of doctors in the hospital setting.

The results of this study showed that there was a significant relationship between the places of residence and their length of stay in a way that those living in rural areas had a longer length of stay than urban residents. Although the number of rural patients admitted to the hospital was less than urban ones, they stayed longer. This could be because they had no proper accommodation in the city. The results of Poorreza et al. [3], Rajaei Fard and Rafiei [8] studies, also the results of Rafiei and Ayatollahi’ research [9] were consistent with the results of the current study. In contrast, Arab et al. [1] in Lorestan hospitals, showed that urban patients stayed longer than rural ones.

However, some studies such as [10–12] resulted that older age was associated factor with prolonged LOS but our study didn’t show a significant relationship (*p* = 0.14).

In this study, patients admitted from other hospitals had longer length of stay, this can be due to the complexity of the illness and worsening of these patients, resulting in the inability of the hospital or the primary care center to treat them. Therefore, they had to be transferred to a pediatric medical center, which can provide first-line care for babies. Of course, the opposite is also true, because the lack of facilities to provide the patient with the services needed may worsen their condition [5], this result confirms with Kandi Kele et al. study [5]. In contrast, in Ravangard et al. study [13], patients who were hospitalized electively had a longer stay than emergency patients. Vahidi et al. [14] showed that the length of stay in emergency patients was longer than elective ones. In the study of Liu et al., [2] patients referred from hospital waiting list and outpatient department, had lower mean LOS.

The results showed that the relationship between the LOS and number of doctor visits, number of counseling, and the number of laboratory tests, number of ultrasonography test were significant. In a study of patients admitted to the emergency department, computed tomography scan or magnetic resonance imaging and intravenous fluids or medications had a significant impact on the length of stay [7].

More laboratory and other diagnostic tests can be ordered by physicians with the aim of better diagnosis and treatment. Also, these tests can be performed due to the severity and complexity of the disease. Therefore, as these tests can be time-consuming, they can affect the length of stay of patients in the hospital. In Ravangard et al’ study [13], para-clinical tests have been shown to increase the length of stay of the patient. Another study resulted in that number of laboratory tests and the number of visits had a significant relationship with LOS [3].

Based on the results, the patient’ LOS is related to the attending physician’s degree, and the cases where their doctor was a sub-specialist have a longer LOS. This is probably due to the type of patients’ disease. In Liu et al. [2] study, the specialty of the doctor was significantly related to the length of stay, and patients treated by oncologists and psychiatrists had longer stays. In the study in the emergency department, there was no likelihood of an increase in the length of stay for patients who were visited or treated by the interns or residents, as compared to attending physicians [7].

This study has several limitations. First, it is a cross-sectional study, so different results may be obtained at different times. Therefore, conducting comparative studies are required at different time’s periods. Second, the study was conducted using the medical records database; therefore, the findings are limited to the available variables in the medical record of patients. So, some influential variables may be excluded. Thirdly, due to the use of data from a single hospital and according to the unique condition of each hospital, there are limitations on the generalization of the results to other centers.

## Conclusion

Factors significantly associated with longer LOS for hospitalized children included the referrals from other hospitals, attending physician’s degree, admission in evening and night, and the number of diagnostic tests. Considering the need to perform some diagnostic services, modifying processes to shorten the process of performing diagnostic tests, providing adequate manpower for specialized services on all hours of the day, and ensuring full patient care, can be taken to reduce the patient’s length of stay. Also, the establishment of a separate and specialized committee in the hospital to evaluate the treatment process in hospitalized patients, regular monitoring of factors affecting the length of stay and expert interventions to accelerate diagnostic and therapeutic processes will be useful**.**

## Data Availability

The datasets used and analyzed during the current study are available from the corresponding author on reasonable request.

## Abbreviations

LOS: Length of stay
IQR: Inter Quartile Range
HIS: Hospital Information System
ICT: Information and Communications Technology
